# Design and Development of a Fully Printed Accelerometer with a Carbon Paste-Based Strain Gauge

**DOI:** 10.3390/s20123395

**Published:** 2020-06-16

**Authors:** Mingjie Liu, Qi Zhang, Yulong Zhao, Yiwei Shao, Dongliang Zhang

**Affiliations:** State Key Laboratory for Manufacturing Systems Engineering, Xi’an Jiaotong University, Xi’an 710049, China; liumingjie@stu.xjtu.edu.cn (M.L.); zhaoyulong@xjtu.edu.cn (Y.Z.); imporeed@stu.xjtu.edu.cn (Y.S.); zhangdl666@stu.xjtu.edu.cn (D.Z.)

**Keywords:** fully printed accelerometer, stereolithography apparatus, screen printing, direct ink writing, low cost, high efficiency

## Abstract

In this paper, we present a fully printed accelerometer with a piezoresistive carbon paste-based strain gauge printed on its surface, which can be manufactured at low cost and with high efficiency. This accelerometer is composed of two parts: a sensor substrate made from high-temperature resin, which is printed by a 3D printer based on stereolithography apparatus (SLA), and a carbon paste-based strain gauge fabricated by screen-printing technology and by direct ink writing (DIW) technology for the purposes of comparison and optimization. First, the structural design, theoretical analysis, simulation analysis of the accelerometer, and analyses of the conductive mechanism and the piezoresistive mechanism of the carbon paste-based strain gauge were carried out. Then the proposed accelerometer was fabricated by a combination of different printing technologies and the curing conditions of the carbon paste were investigated. After that, the accelerometers with the screen-printed strain gauge and DIW strain gauge were characterized. The results show that the printing precision of the screen-printing process on the sensor substrate is higher than the DIW process, and both accelerometers can perform acceleration measurement. Also, this kind of accelerometer can be used in the field of measuring body motion. All these findings prove that 3D printing technology is a significant method for sensor fabrication and verification.

## 1. Introduction

Three-dimensional printing technology is a family of layer-by-layer fabrication processes for the fabrication of objects, which has the advantages of low cost, high efficiency, simple process steps, the ability to fabricate complex structures and a wide range of materials for selection [1,2,3]. Some typical examples of 3D printing technologies are stereolithography apparatus (SLA), fused deposition modeling (FDM), selective laser sintering (SLS), direct ink writing (DIW) and inkjet printing [4,5]. Among them, SLA is the oldest method and one of the most popular 3D printing technologies since it was firstly reported by Charles W. Hull in 1986 as a tool to fabricate 3D structures [6].

During the SLA process, the plastic monomers are directly shaped by the photopolymerization process and based on the 3D computer-aided design (CAD) model to form complex structures [7]. Due to its high accuracy, simple process and low cost, SLA has been widely used in fields such as bioengineering, micro electro mechanical systems (MEMS), and electronic and mechanical devices [8]. In recent years, with the boom in 3D printing technology, 3D printing has become an alternative means of fabricating mechanical quantity sensors, such as pressure sensors [9,10,11], strain sensors [12,13,14] and force sensors [15,16,17]. As an important 3D printing technology, SLA technology has also become the focus of research in the field of mechanical quantity sensor fabrication [18,19,20,21].

A mechanical quantity sensor is usually composed of three parts: the sensing element, conversion element and measuring circuit. Normally, the sensing element is non-conductive while the conversion element and measuring circuit are conductive. Therefore, multi-materials are needed in the mechanical quantity sensor fabrication process. However, the conductive resin for the SLA process is still in the laboratory stage and has not been commercialized [22,23], and SLA-based printers that are capable of multi-materials printing are rare and expensive [24]. In order to realize the low cost and highly efficient manufacturing of mechanical quantity sensors, it is important to study a way of combining SLA technology with other printing technologies. Although there is current research on the combination of SLA technology with screen printing technology [21] or inkjet printing technology [25] to fabricate low cost fully printed sensors, to the best of our knowledge, such studies are still relatively rare and need to be extended.

In view of these above points, a fully printed accelerometer is proposed in this paper that utilizes a combination of different printing technologies. During fabrication, the sensor substrate was printed by an SLA-based printer using a high-temperature resin, and a carbon paste-based strain gauge was printed on the surface of the sensor substrate by screen printing or DIW technologies, and were compared to each other. Then, the morphology of the strain gauges made by the two different methods were tested to choose a suitable way to fabricate the strain gauge on the resin substrate. Also, the conductive mechanism and the piezoresistive mechanism of the carbon paste-based strain gauge were elucidated from a microscopic point of view, and the effects of the curing conditions on the resistance and piezoresistive properties of the carbon paste-based strain gauge were investigated. Finally, several preliminary tests were carried out in order to verify the function of the accelerometer.

This paper supplements the research on combining SLA technology with other printing technologies, such as screen-printing technology and DIW technology, to fabricate low cost fully printed sensors. Additionally, the method described in this paper is very useful to improve the integration of the sensor structure because the accelerometer was fully printed. Also, the accelerometer can be fabricated in a few hours and the processing cost is very low due to the use of the desktop consumer-level 3D printer. That also means it is a quick and easy way to get from a sensor design to a sensor prototype. All in all, this work provides a cost-effective and highly efficient way to fabricate accelerometers.

## 2. Materials and Methods

### 2.1. Sensor Design and Analysis

#### 2.1.1. Structure Design and Theoretical Analysis of the Accelerometer

**Figure 1:**
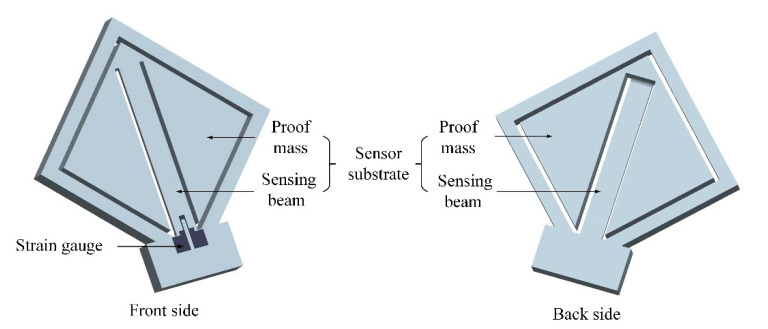
Diagrammatic sketch of the proposed accelerometer.

**Figure 2:**
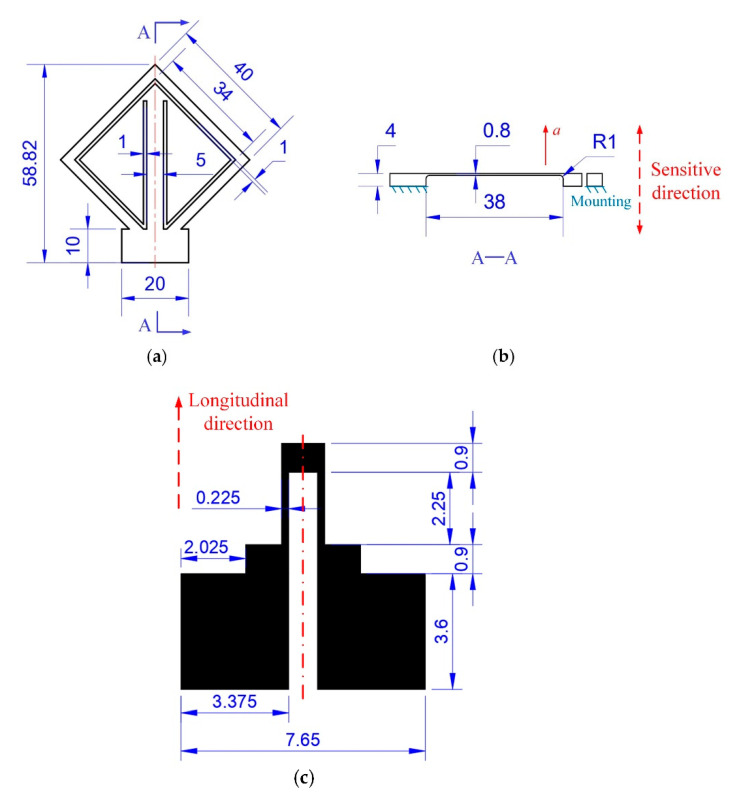
Dimensions of the sensor substrate: (**a**) top view; and (**b**) sectional view. (**c**) Dimensions of the strain gauge. Units: mm.

The diagrammatic sketch of the proposed accelerometer is shown in Figure 1. The accelerometer consists of a sensor substrate and a strain gauge printed on the surface of it. The sensor substrate is mainly composed of a proof mass and a sensing beam. One end of the sensing beam is connected to the proof mass to suspend it, and the strain gauge is located at the other end of the sensing beam. To get the maximum length, the sensing beam is arranged diagonally to the sensor frame. The dimensions of the sensor substrate and the strain gauge are shown in Figure 2. The design and analysis of this strain gauge are presented in our previous work [26], which found that the effective gauge factor of this strain gauge was 0.91 times as much as the intrinsic gauge factor of the raw material.

As shown in Figure 2b, when an acceleration *a* is applied along the direction normal to the sensor substrate, i.e., the *z* axis, the proof mass will move vertically due to the inertial force, so, the sensing beam attached to it will deform. In this case, the sensing beam approximately acts as a fixed-guided beam, and it is subjected to the inertial force from the proof mass, which can be expressed as:

(1) F=ma,

where *F* is the inertial force from the proof mass, and *m* is the mass of the proof mass.

The maximum longitudinal strain in the sensing beam induced by the inertial force occurs at the ends of the beam, which can be written:

(2) $$\varepsilon_{l\max}=\frac{Flt}{4EI},$$

where

(3) $$I=\frac{wt^{3}}{12},$$

where *l*, *w* and *t* are the length, width and thickness of the sensing beam, respectively; *ε_l_*_max_ is the maximum longitudinal strain; *E* is the Young’s modulus of the sensing beam, and *I* is the moment of inertia with respect to the neutral axis. Substituting Equations (1) and (3) into Equation (2), the maximum longitudinal strain in the sensing beam can be expressed as:

(4) $$\varepsilon_{l\max}=\frac{3ml}{Ewt^{2}}a.$$

Then the relative change of resistance of the strain gauge caused by the applied acceleration can be obtained [27]:

(5) $$\frac{\Delta R}{R_{0}}=G\varepsilon_{l\max}=G\frac{3ml}{Ewt^{2}}a,$$

where *R*_0_ is the initial resistance value of the strain gauge without the load; Δ*R* is the change of *R*_0_; and *G* is the effective gauge factor of the strain gauge.

**Figure 3:**
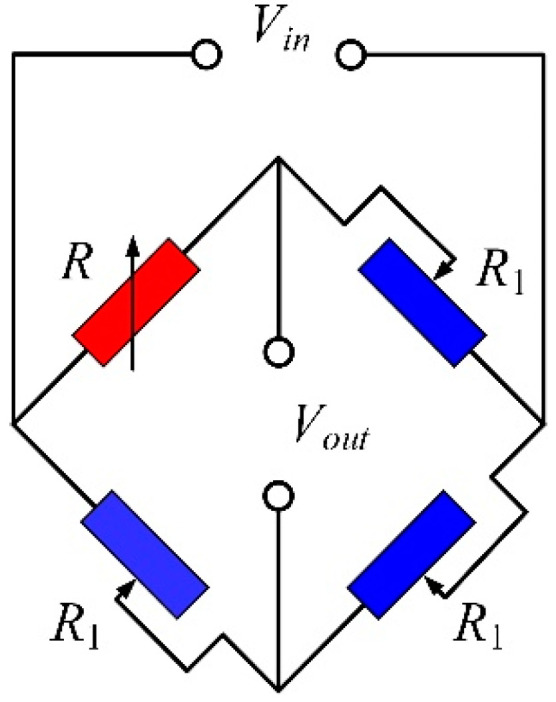
Schematic of the Wheatstone bridge circuit used in the proposed accelerometer.

As shown in Figure 3, resistance changes are often read using the Wheatstone bridge circuit configuration. When the strain gauge and the other three adjustable resistors form a single arm Wheatstone bridge circuit, the output voltage of the Wheatstone bridge circuit can be written as [28]:

(6) $$V_{out}=\frac{R_{1}R-R_{1}^{2}}{2R_{1}(R+R_{1})}\approx \frac{\Delta R}{4R_{0}}V_{in},$$

where *R* is the resistance of the strain gauge with the load and *R* = *R*_0_ + Δ*R*; *R*_1_ is the resistance of the adjustable resistors and adjusts *R*_1_ to be equal to *R*_0_; and *V_out_* and *V_in_* are the output voltage and the input voltage of the Wheatstone bridge circuit, respectively. Then, substituting Equation (5) into Equation (6), the output voltage of the accelerometer can be obtained:

(7) $$V_{out}=G\frac{3ml}{4Ewt^{2}}aV_{in}.$$

Equation (7) indicates that the output voltage of the accelerometer and the applied acceleration are linearly related to each other. Therefore, by measuring the output voltage of the accelerometer, the value of the acceleration to be measured can be obtained.

#### 2.1.2. Simulation Analysis of the Accelerometer

**Figure 4:**
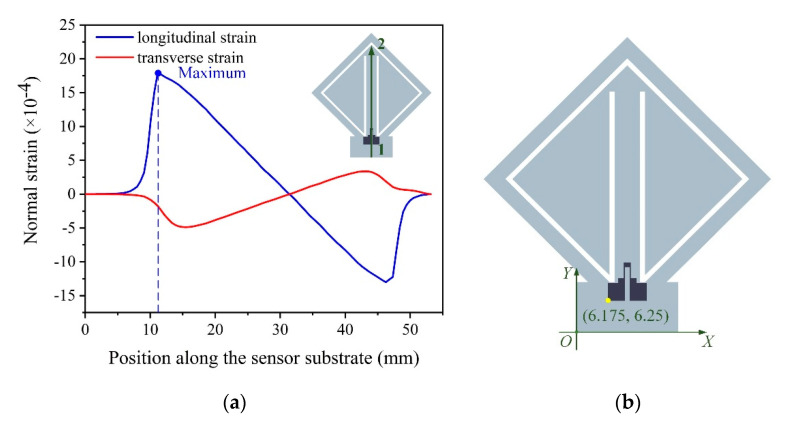
(**a**) The longitudinal strain and the transverse strain distributions along the path from point 1 to point 2 on the sensor substrate. (**b**) The position of the strain gauge on the sensor substrate.

To determine the proper position of the strain gauge on the sensor substrate, the normal strain distribution along the sensor substrate under the effect of the acceleration *a* was analyzed in the software, ANSYS Workbench 14.5 (Ansys Inc., Canonsburg, PA, USA) using the finite element method (FEM). The elastic modulus of the sensor substrate *E* = 2.75 GPa and the Poisson ratio of the sensor substrate *ν* = 0.35. During the simulation process, a 4 g acceleration was applied to the accelerometer with the direction normal to the sensor substrate. The simulation results show that the maximum equivalent stress with a value of 5.4497 MPa occurs at the fixed end of the sensing beam, which is much less than the flexural strength of the sensor substrate, 94.5 MPa. The longitudinal strain and the transverse strain distributions along the path from point 1 to point 2 on the sensor substrate are shown in Figure 4a. As shown in Figure 4a, near the fixed end of the sensing beam, the longitudinal strain is much larger than the transverse strain, so the transverse strain has a tiny influence on the resistance change of the strain gauge. In order to make the accelerometer have a larger output, the strain gauge should be put at the place where the normal strain is large, so the position of the strain gauge was determined as shown in Figure 4b, which is (6.175, 6.25).

#### 2.1.3. Conductive Mechanism and Piezoresistive Mechanism of the Carbon Paste-Based Strain Gauge

The strain gauge proposed in this paper was made from carbon paste (CP-1000, Guangdong Nanhai ETEB Technology Co., Ltd., Foshan, China). This carbon paste mainly consists of carbon black as the filler, unsaturated polyester resin (UPR) as the polymer matrix, organic solvent and isocyanate curing agent. During the heating process, the organic solvent evaporates and the UPR cures under the action of the isocyanate curing agent. Eventually, a stable carbon paste-based polymer matrix composite is formed. So, the conductive mechanisms for conductive polymer matrix composites, such as percolation and tunneling, can be applied to explain the conductive and piezoresistive properties of this kind of carbon paste-based strain gauge. In practice, both conductive mechanisms can take place simultaneously within such polymer matrix composites, and depending on the internal structure of the composites, one of them may dominate [29,30].

**Figure 5:**
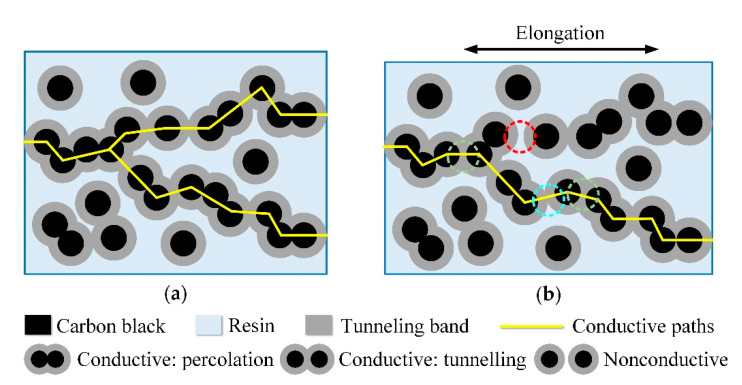
The conductive mechanism and piezoresistive mechanism of the carbon paste-based strain gauge from a micro perspective: (**a**) Without the load; (**b**) Being stretched. The red, blue and green dashed circles emphasize the changes in the spacing between carbon black particles.

As shown in Figure 5, when the carbon black particles (the black circles) are in contact with each other, the conductivity between the carbon black particles can be elucidated by percolation. When the carbon black particles are non-touching, but the space between them is relatively small (in the range of nm) and within the tunneling band (the grey circular ring around the black circle), the electrons can penetrate through the resin in the interspace so the conductivity between the carbon black particles can be elucidated by tunneling. When the carbon black particles are non-touching, but the spacing between them is relatively large and the tunneling current between the carbon black particles is difficult to generate, it forms non-conduction zones. The percolation and tunneling inside the carbon paste-based strain gauge creates the conductive paths, so the carbon paste-based strain gauge is electroconductive. During the process, strain occurs and the strain gauge is stretched. From a micro perspective, it increases the distances between the internal carbon black particles. Hence, it will cause the particles that were previously conductive to no longer be conductive due to the larger spacing (as shown in the red dashed circle in Figure 5), and cause the tunnel current between the particles, which previously had the tunnel effect, to be smaller due to the larger spaces (as shown in the blue dashed circle in Figure 5). Also, carbon black particles that originally contacted each other and percolated turn to tunneling because the larger distances keep them from contacting each other (as shown in the green dashed circles in Figure 5). All these factors reduce the number of the conductive paths or increase the resistance of the original conductive paths inside the carbon paste-based strain gauge. So, at a macro level, the resistance of the strain gauge becomes larger. This is the microscopic explanation of the piezoresistive properties of this kind of carbon paste-based strain gauge.

### 2.2. Sensor Fabrication

First, the sensor substrate was fabricated with the use of SLA-based 3D printing technology. The equipment used in this process is an SLA-based desktop consumer-level 3D printer Form 2 from Formlabs (Somerville, MA, USA), with key parameters including a laser facula diameter of 140 µm and a thickness resolution up to 25 µm. The raw material used in this process is a transparent high-temperature resin, which is also from Formlabs (Somerville, MA, USA). The heat deflection temperature (HDT) of this high-temperature resin is 238 °C at 0.45 MPa, and other parameters of this high-temperature resin are shown in Table 1 [31]. During the printing process, each printed layer of the high-temperature resin was 100 µm thick. In order to ensure that the sensor substrate has better morphology and mechanical properties, and that it is compatible with the subsequent screen printing and DIW processes, some post processing is needed. First, the sensor substrate was ultrasonically cleaned successively in isopropanol and ethanol to remove resin residues. Then, the sensor substrate was post-cured in an ultraviolet (UV) curing machine, Form Cure from Formlabs (Somerville, MA, USA) at 60 °C for 60 min to ensure that the sensor substrate acquired the preferred mechanical properties, as shown in Table 1. Finally, the sensor substrate was polished by the polishing plates to further remove the resin residue and reduce surface roughness.

**Figure 6:**
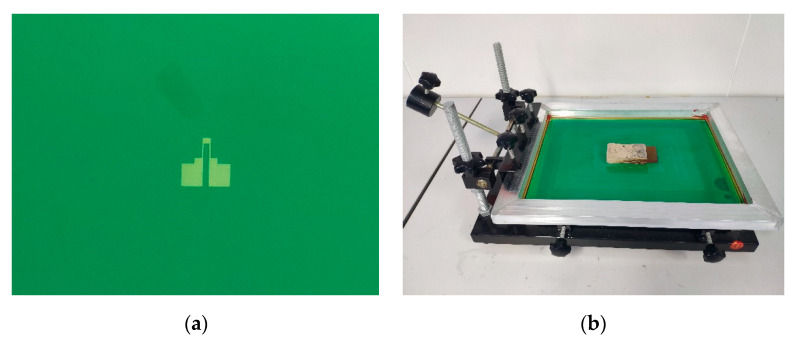
Photographs: (**a**) the screen mask with the pattern of the strain gauge on it; and (**b**) the manual screen-printing machine.

**Figure 7:**
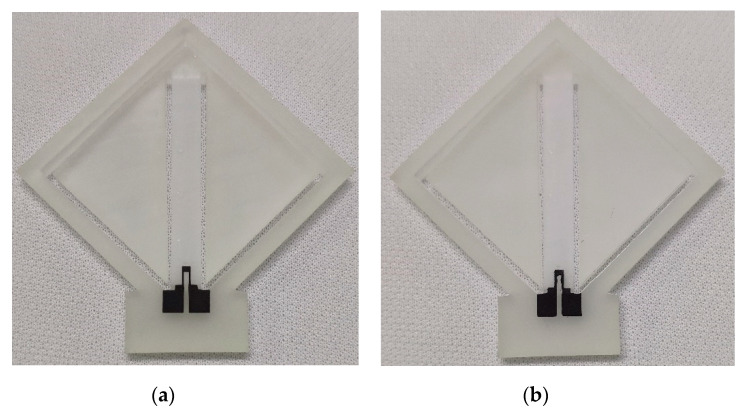
Photographs of (**a**) the accelerometer with the screen-printed strain gauge; and (**b**) the accelerometer with the direct ink writing (DIW) strain gauge.

Then, the strain gauge was printed on the surface of the sensor substrate with the use of screen-printing technology and direct ink writing technology. As mentioned above, both the screen-printed strain gauge and the DIW strain gauge were made from the same carbon paste. As for the screen-printing process, the screen mask was made by ETBT (Guangdong Nanhai ETEB Technology Co., Ltd., China). The parameters of this screen mask are 300 mesh, 45° stretch angle and 10 µm film thickness, as shown in Figure 6a. Because the strain gauge pattern is relatively simple and in order to reduce production costs, the screen printing was processed on a low cost, manual screen-printing machine, as shown in Figure 6b. After printing, in order to evaporate the solvent completely and cure the paste to make the strain gauge functional, the sensor substrate with the strain gauge was dried in an air convection oven at 120 °C for 30 min. Because the drying temperature (120 °C) is much lower than the HDT of the high-temperature resin (243 °C), no thermal deformation occurs on the sensor substrate. The accelerometer with the screen-printed strain gauge is shown in Figure 7a.

**Figure 8:**
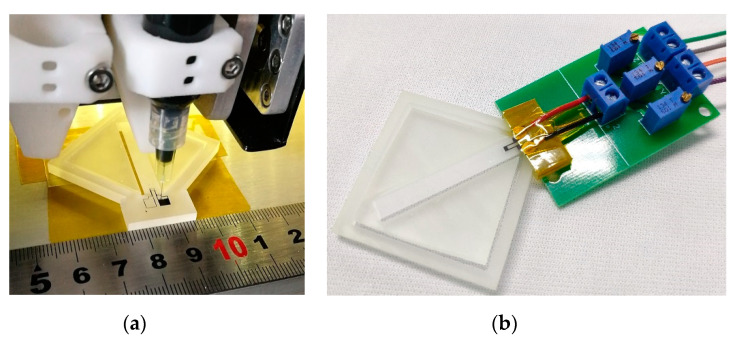
Photographs of (**a**) the strain gauge during the DIW process; and (**b**) the fabricated accelerometer with the measuring circuit.

During the direct ink writing process, the strain gauge was fabricated by a pneumatic DIW based 3D printer (JD200 Pro, Xi’an Ruite 3D Technology Co., Ltd., Xi’an, China) with ±50 µm printing resolution and a pinhole diameter of 100 µm. The strain gauge during the DIW process is shown in Figure 8a. The post processing of the DIW strain gauge was the same as the process used for the screen-printing process. The accelerometer with the DIW strain gauge is shown in Figure 7b.

Finally, the printed accelerometers were bonded with wires to enable access to the external circuit, which mainly consists of three adjustable resistors that constitute the single arm Wheatstone bridge circuit. The fabricated accelerometer with the measuring circuit is shown in Figure 8b.

## 3. Results and Discussion

### 3.1. Characterization of the Carbon Paste-Based Strain Gauge

**Figure 9:**
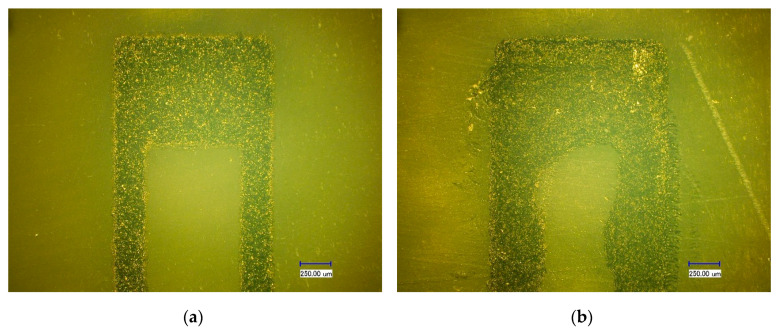
Microscope photos of (**a**) the screen-printed strain gauge; and (**b**) the DIW strain gauge.

First, the screen-printed strain gauge and DIW strain gauge were characterized. The photos of these strain gauges taken by a digital microscope (VH-8000, KEYENCE, Osaka, Japan) with 100 times magnification are shown in Figure 9. The morphology of the screen-printed strain gauge is better than the DIW strain gauge. The edge of the screen-printed strain gauge is clear and straight, while the inner edge of the DIW strain gauge is almost serpentine. The width of the screen-printed strain gauge longitudinal branches is 251.67 ± 7.02 μm, and the width of the DIW strain gauge longitudinal branches is 366.10 ± 49.79 μm. Compared to the width of the longitudinal branches of the design, which is 225 μm, the printing precision of the screen-printing process on the resin sensor substrate is better than the DIW process. This is because the hand-polished resin sensor substrate is still not a strictly flat surface, which affects the spacing between the needle and the sensor substrate during the DIW process. So, the uneven sensor substrate has a great influence on the DIW process, however, screen printing is a contact printing method and the uneven sensor substrate has less impact on the screen-printing process. This proved that compared with DIW technology, screen-printing technology is more suitable for printing strain gauges on resin substrates that are not very flat. Also, the resistance of the screen-printed strain gauge and the DIW strain gauge was measured by a FLUKE 8846A 6-1/2 digital precision multimeter (Fluke Corporation, Everett, WA, USA), and this was 5.13607 kΩ and 1.69073 kΩ, respectively.

### 3.2. Investigation of the Piezoresistive Properties of the Carbon Paste-Based Strain Gauge

**Figure 10:**
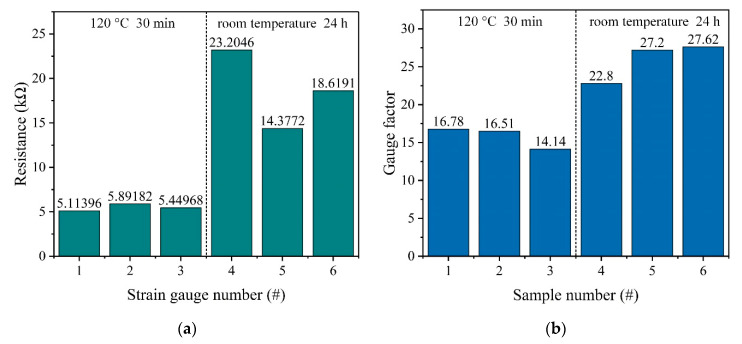
The results of (**a**) the resistance measurement; and (**b**) the three-point bending test.

Because the carbon paste-based strain gauge need to be dried in an air convection oven to evaporate the solvent completely and to cure the paste to make it functional, the influence of the curing conditions on the resistance and piezoresistive properties of the carbon paste-based strain gauge was investigated. First, six strain gauges designed as shown in Figure 2c were screen printed on the polished resin substrate. Then, strain gauges #1~#3 were dried in an air convection oven at 120 °C for 30 min to cure the paste, while strain gauges #4~#6 were dried at room temperature for 24 h. After that, the resistances of these strain gauges were measured by a FLUKE 8846A 6-1/2 digital precision multimeter (Fluke Corporation, Everett, WA, USA), and the results of the resistance measurement are shown in Table 2 and Figure 10a.

**Figure 11:**
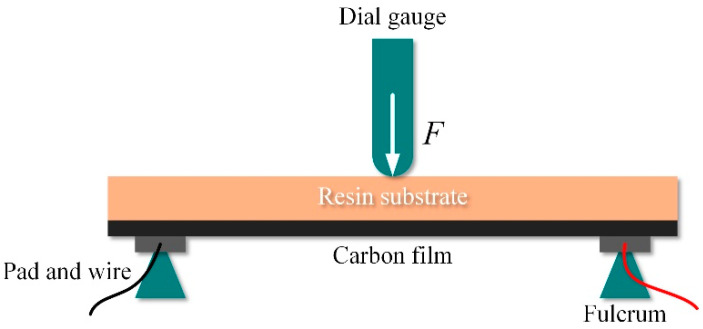
The schematic diagram of the three-point bending test.

Then, six rectangular text samples made from the high-temperature resin that were 25 mm long, 5 mm wide and 0.5 mm thick were prepared by the SLA-based printing process. Then the carbon paste was screen printed on the entire upper surface of the polished samples. Next, to cure the paste, samples #1~#3 were dried in an air convection oven at 120 °C for 30 min, while samples #4~#6 were dried at room temperature for 24 h. Finally, the three-point bending test was carried out to measure the gauge factor of these samples, as shown in Figure 11. The gauge factor of the sample can be calculated by the equation [32,33]:

(8) G=l23tΔYΔRR0=l23tΔRR0ΔY,

where *l* is the distance between two fulcrums; *t* is the thickness of the sample; Δ*Y* is the change in midpoint vertical positioning after loading; Δ*R* is the change in resistance of the sample, and *R*_0_ is the initial resistance of the sample.

Equation (8) can be divided into two parts, *l*^2^/3*t* is calculated by the dimensions of the samples and the experimental setup, and (∆*R*/*R*_0_)/∆*Y* is calculated by the experimental data recorded. In the experiment, in order to ensure the accuracy of the results, the distance between two fulcrums was supposed to be the same as the distance between two wires on the sample. During the test, loads were applied to the center of each sample and caused a vertical deformation from 0 mm to about 0.7 mm in this position. The vertical deformation of the sample center was measured by a dial gauge (NSCING, Nanjing, China) and the resistance change in the sample was simultaneously recorded by a FLUKE 8846A 6-1/2 digital precision multimeter (Fluke Corporation, Everett, WA, USA). Then the gauge factor of each sample was calculated. The results of the three-point bending test are shown in Table 3 and Figure 10b.

**Figure 12:**
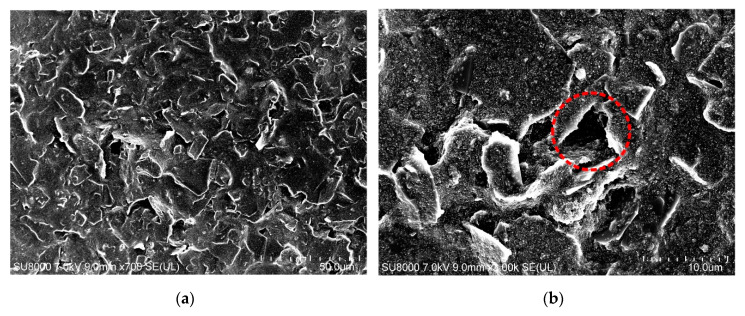

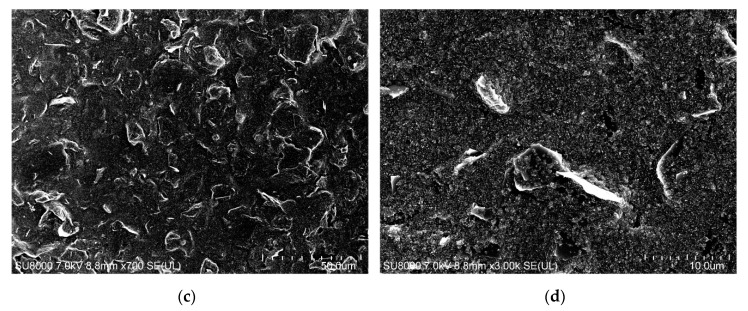
Scanning electron microscope images of the carbon paste films: (**a**) dried in an air convection oven at 120 °C for 30 min, with 700 times magnification; (**b**) dried in an air convection oven at 120 °C for 30 min, with 3k times magnification; (**c**) dried at the room temperature for 24 h, with 700 times magnification; and (**d**) dried at the room temperature for 24 h, with 3k times magnification. The cavity is highlighted by the red dashed circle.

As shown in Figure 10a, the resistance of the strain gauges dried in an air convection oven at 120 °C for 30 min is 5.49 ± 0.68 kΩ, while the resistance of the strain gauges dried at room temperature for 24 h is 18.73 ± 7.65 kΩ. So, the resistance of the strain gauges dried in an air convection oven at 120 °C for 30 min is smaller than the resistance of the strain gauges dried at room temperature for 24 h. Also, as shown in Figure 10b, the gauge factor of the samples dried in an air convection oven at 120 °C for 30 min is smaller than the gauge factor of the samples dried at room temperature for 24 h, which are 15.81 ± 2.52 and 25.87 ± 4.62, respectively. Because the gauge factor of the carbon paste-based strain gauge is 0.91 times as much as the intrinsic gauge factor of the carbon paste, i.e., the gauge factor of the carbon paste-based samples, the gauge factor of the carbon paste-based strain gauges dried in an air convection oven at 120 °C for 30 min and dried at the room temperature for 24 h are 14.39 ± 2.29 and 23.54 ± 4.20 respectively. To better understand these results, the morphology of carbon paste films dried in an air convection oven at 120 °C for 30 min and dried at room temperature for 24 h were investigated by the use of a scanning electron microscope SU-8010 from HITACHI (Tokyo, Japan). As shown in Figure 12, the carbon paste film dried in an air convection oven at 120 °C for 30 min is denser, while the carbon paste film dried at room temperature for 24 h is poriferous. The cavities in the carbon paste film may be caused by the non-uniform volatilization of the solvent in the carbon paste at room temperature. Also, it is difficult for the solvent to completely volatilize at room temperature, which leads to an decrease in the volume fraction of carbon black in the carbon paste film and an increase in the average distance between the carbon black particles. All these factors lead to a larger resistance and a larger standard deviation of resistance in the strain gauges dried at room temperature. In addition, different curing temperature conditions will affect the mechanical properties of the UPR after curing [34]. Because the curing temperature was not met, the curing rate of the UPR in the carbon paste film dried at room temperature is lower, which also results in a lower Young’s modulus (*E*) of the UPR. In the carbon paste film, the carbon black is much stiffer than the UPR and in the carbon paste film dried at room temperature, the difference between the *E* of the UPR and the *E* of the carbon black is even greater. According to the theory proposed by Grimaldi et al. [35], when the carbon paste-based strain gauge dried at room temperature is stretched, the strain will be more concentrated on the UPR compared to the carbon paste-based strain gauge dried at 120 °C for 30 min. This results in a larger variation in the space between the carbon black particles, which makes the gauge factor of the carbon paste-based strain gauge dried at room temperature larger. Although the gauge factor of the carbon paste-based strain gauge dried at room temperature is larger, which is beneficial to the sensitivity improvement of the accelerometer, the standard deviation of resistance of this carbon paste-based strain gauge is also larger, which weakens the process consistency. Therefore, in the process described in this paper, the carbon paste-based strain gauge is dried in an air convection oven at 120 °C for 30 min to cure the carbon paste.

### 3.3. Characterization of the Accelerometer

**Figure 13:**
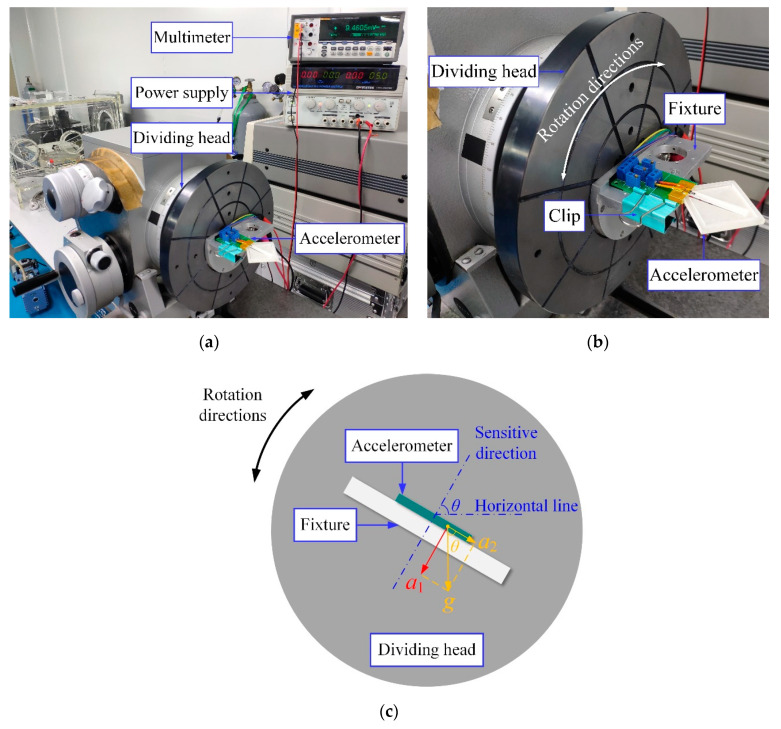
(**a**) The experimental setup for the tumbling experiments. (**b**) The details of the precise dividing head with the accelerometer on it. (**c**) The schematic diagram of the tumbling experiments.

Firstly, tumbling experiments were conducted to evaluate the static characteristics of the accelerometers. As shown in Figure 13a, the experimental setup consists of a precise dividing head (SJJF-1, THJD, Shanghai, China) with a resolution of 1″, a DC stabilized power supply (GPS-3303C, GW Instek, Taiwan, China) to input 5V DC voltage to the Wheatstone bridge circuit in the accelerometer and a digital multimeter (FLUKE 8846A 6-1/2, Fluke Corporation, Everett, WA, USA) to record the output voltage of the Wheatstone bridge circuit in the accelerometer. As shown in Figure 13c, during the test, when the dividing head is rotated in the earth gravitational field and *θ* changes from −90° to 90°, the acceleration applied to the accelerometer can be divided into two parts, *a*_1_ and *a*_2_, which can be expressed as [36]:

(9) $$\left\{ \begin{array}{l} a_{1}=g\sin\theta \\ a_{2}=g\cos\theta \end{array}\right.,$$

where *g* is the gravitational acceleration and *θ* is the angle between the sensitive direction of the accelerometer and the horizontal line. Thus, the dividing head can apply an acceleration *a*_1_ in the sensitive direction of the sensor, which changes from −1 g to 1 g.

**Figure 14:**
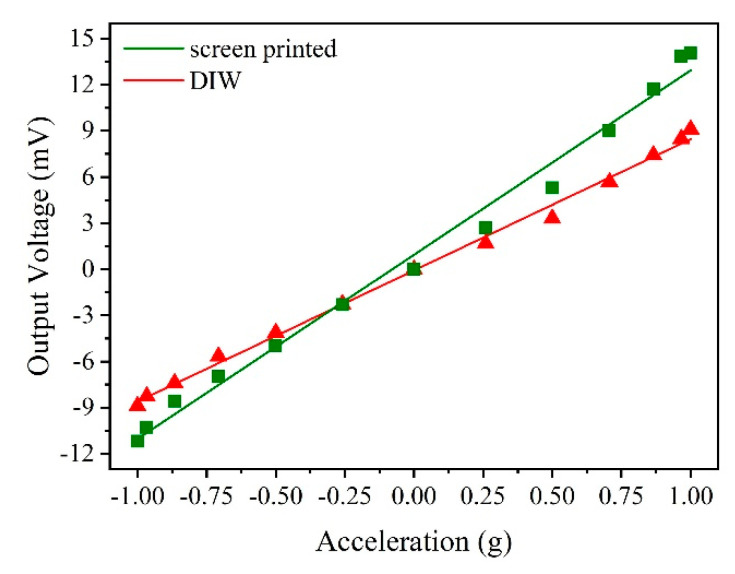
Experimental results of the tumbling experiments.

The experimental results are shown in Table 4 and Figure 14. The sensitivities of the accelerometers with the screen-printed strain gauge and DIW strain gauge were 11.98572 mV/g and 8.53491 mV/g, respectively. These results indicate that both of the accelerometers are able to fulfil the function of acceleration measurement and the accelerometer with the screen-printed strain gauge has a higher sensitivity than the accelerometer with the DIW strain gauge.

**Figure 15:**
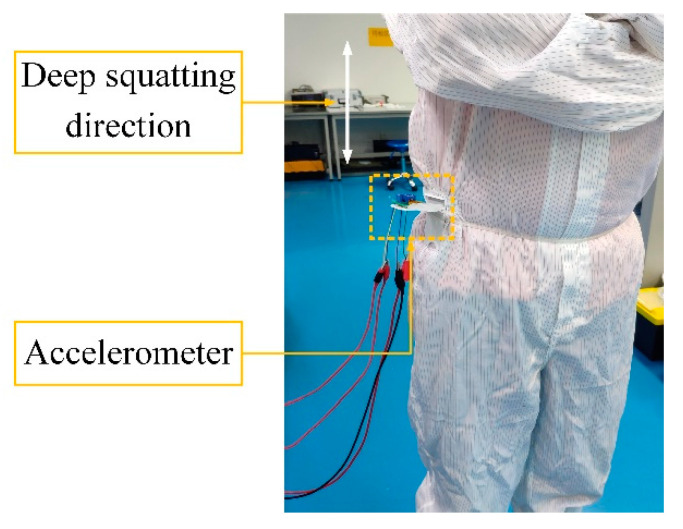
Accelerometer installation for the body motion measurements.

**Figure 16:**
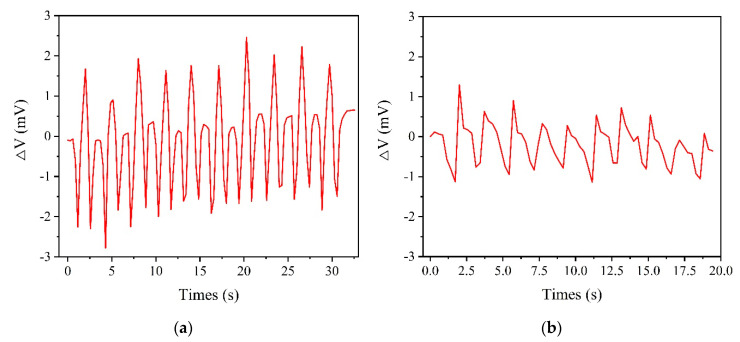
(**a**) The change in output voltage of the accelerometer during 10 deep-squatting cycles. (**b**) The change in output voltage of the accelerometer while walking 20 steps in place.

Then, the accelerometer with the screen-printed strain gauge was attached to the hip joint of the tester and the sensitive direction of the accelerometer was perpendicular to the ground, as shown in Figure 15. The tester deep-squatted 10 times and walked 20 steps (10 steps for each foot) in place and the change in the output voltage of the accelerometer during the two actions was recorded, as shown in Figure 16. The accelerometer was able to respond to the movements of the human body and the output voltage variation curve of the accelerometer during the deep-squatting cycles is different from the walking periods. The consistency in the change of output voltage during the 10 deep-squatting cycles is good, and the slight drifting in the output voltage variation curve may be caused by creepage of the sensor substrate. The results show that the movements of the tester can be detected and this 3D printed accelerometer may be used to measure body motion and in related fields.

### 3.4. Discussion

A fully printed carbon paste-based accelerometer based on a combination of different printing technologies is presented in this paper. Although both screen printing and DIW were able to fabricate carbon paste-based strain gauges, the screen-printing process has better precision on the resin substrate than the DIW process. This is because the uneven surface of the resin substrate has less impact on the screen-printing process. However, it is necessary to study both processes because DIW is a maskless process that is easy to adjust and suitable for producing sensor prototypes, while the screen-printing process is suitable for the mass production of sensors. This study was a preliminary exploration of the application of these two processes in the field of 3D printed mechanical quantity sensors.

We combined a specific carbon paste with conductive composite material theory [29,30] and gauge factor enhancement theory of thick film resistors [35] to explain the conductive mechanism and piezoresistive mechanism of the carbon paste-based strain gauge, and investigated the effect of the curing temperature conditions on the resistance and gauge factor of the carbon paste-based strain gauge. The results show that the curing temperature conditions have an impact on both the resistance and the gauge factor of the carbon paste-based strain gauge. The gauge factor of the carbon paste-based strain gauge dried at room temperature is larger and the standard deviation of the resistance is also larger. In the future, it is necessary to study the influence of the curing temperature and curing time on the resistance and the gauge factor of the carbon paste-based strain gauge, to obtain a way to improve the gauge factor of this kind of strain gauge to ensure the consistency of the resistance. This is conducive to eventually improving the performance of the sensor.

Because this paper mainly focuses on the basic research and process study of the printing technologies, only preliminary tests were performed on the proposed accelerometer. The performance of the accelerometer can be further improved. More detailed tests and optimization of the accelerometer are needed in the future.

## 4. Conclusions

Using a combination of different printing technologies, this paper presents a fully printed accelerometer with a piezoresistive carbon paste-based strain gauge that can be produced in a cost-effective and highly efficient way. The accelerometer consists of a sensor substrate that is printed by a desktop 3D printer based on SLA using high-temperature resin, and a carbon paste-based strain gauge fabricated by screen-printing technology or DIW technology. In this work, the accelerometer was theoretically analyzed and then verified through FEM. The conductive mechanism and the piezoresistive mechanism of the carbon paste-based strain gauge were explained from a microscopic point of view. Next, the proposed accelerometer was fabricated by different printing technologies. After that, the screen-printed strain gauge and DIW strain gauge were each characterized to find a suitable way to fabricate the strain gauge on the resin substrate. In addition, the influence of the curing conditions on the resistance and piezoresistive properties of the carbon paste-based strain gauge was investigated. Finally, some preliminary tests were conducted to characterize these accelerometers. The results show that the printing precision of the screen-printing process on the resin sensor substrate is higher than the DIW process, and both accelerometers can perform the function of acceleration measurement. Also, this 3D printed accelerometer can be used in the field of body motion measurements. All these results prove that 3D printing technology is a promising way to fabricate sensors and it provides a cost-effective and highly efficient method to fabricate fully printed sensors.

## Figures and Tables

**Table 1:** Properties of the high-temperature resin.

Properties	Green ^1^	Post-Cured ^2^	Method
Ultimate tensile strength	20.9 MPa	58.3 MPa	ASTM D 638-14
Elongation at break	14%	3.3%	ASTM D 638-14
Tensile modulus	0.75 GPa	2.75 GPa	ASTM D 638-14
Flexural strength at break	24.1 MPa	94.5 MPa	ASTM D 790-15
Flexural modulus	0.69 GPa	2.62 GPa	ASTM D 790-15
Thermal expansion (0–150 °C)	118.1 (μm/m/°C)	79.6 (μm/m/°C)	ASTM E 831-13

^1^ Data was obtained from green parts, printed using Form 2100 μm, High Temp settings, washed for 5 min in Form Wash and air dried without post cure. ^2^ Data was obtained from parts printed using a Form 2100 μm, High Temp settings, and post-cured with Form Cure at 60 °C for 60 min.

**Table 2:** Data for the resistance of the carbon paste-based strain gauges.

Strain Gauge (#)	Resistance (kΩ)
1	5.11396
2	5.89182
3	5.44968
4	23.2046
5	14.3772
6	18.6191

**Table 3:** Data for the gauge factor of the carbon paste-based samples.

Test Sample (#)	Gauge Factor
1	16.78
2	16.51
3	14.14
4	22.80
5	27.20
6	27.62

**Table 4:** Data from the tumbling experiments.

Angle *θ* ^1^ (°)	Acceleration (g)	ΔV (mV)	
		Screen Printed	DIW
−90	−1	−11.2048	−8.8877
−75	−0.966	−10.2810	−8.2614
−60	−0.866	−8.5833	−7.4040
−45	−0.707	−7.0026	−5.6695
−30	−0.5	−4.9683	−4.1343
−15	−0.259	−2.3217	−2.2670
0	0	0	0
15	0.259	2.6855	1.6702
30	0.5	5.2875	3.3052
45	0.707	9.0066	5.6687
60	0.866	11.7177	7.4244
75	0.966	13.8422	8.4762
90	1	14.0693	9.0919

^1^ The angle *θ* is between the sensitive direction of the accelerometer and the horizontal line.

## References

[1] Chua C.K., Yeong W.Y., An J. (2017). 3D Printing and Bioprinting in MEMS Technology. Micromachines.

[2] Lind J.U., Busbee T.A., Valentine A.D., Pasqualini F.S., Yuan H., Yadid M., Park S.J., Kotikian A., Nesmith A.P., Campbell P.H. (2017). Instrumented Cardiac Microphysiological Devices via Multimaterial Three-Dimensional Printing. Nat. Mater..

[3] Leal-Junior A., Casas J., Marques C., Pontes M.J., Frizera A. (2018). Application of Additive Layer Manufacturing Technique on the Development of High Sensitive Fiber Bragg Grating Temperature Sensors. Sensors.

[4] Sharafeldin M., Jones A., Rusling J.F. (2018). 3D-Printed Biosensor Arrays for Medical Diagnostics. Micromachines.

[5] Lan H.B., Li D.C., Lu B.H. (2015). Micro and Nano 3D Printing. Sci. Sin. Tech..

[6] Hull C. (1986). Apparatus for Production of Three-Dimensional Objects by Stereolithography. U.S. Patent.

[7] Chockalingam K., Jawahar N., Chandrasekar U., Ramanathan K.N. (2008). Establishment of Process Model for Part Strength in Stereolithography. J. Mater. Process. Tech..

[8] Bertana V., De Pasquale G., Ferrero S., Scaltrito L., Catania F., Nicosia C., Marasso S.L., Cocuzza M., Perrucci F. (2019). 3D Printing with the Commercial UV-Curable Standard Blend Resin: Optimized Process Parameters towards the Fabrication of Tiny Functional Parts. Polymers.

[9] Emon M.O.F., Choi J.-W. (2017). Flexible Piezoresistive Sensors Embedded in 3D Printed Tires. Sensors.

[10] Kim H., Torres F., Wu Y.Y., Villagran D., Lin Y.R., Tseng T.L. (2017). Integrated 3D Printing and Corona Poling Process of PVDF Piezoelectric Films for Pressure Sensor Application. Smart Mater. Struct..

[11] Tuna A., Erden O.K., Gokdel Y.D., Sarioglu B. 3D Printed Capacitive Pressure Sensor with Corrugated Surface. Proceedings of the 13th Conference on Ph.D. Research in Microelectronics and Electronics (PRIME)/14th International Conference on Synthesis, Modeling, Analysis and Simulation Methods and Application to Circuit Design (SMACD).

[12] Seo M., Hwang S., Hwang T., Yeo J. (2019). Fabrication of Soft Sensor Using Laser Processing Techniques: For the Alternative 3D Printing Process. Materials.

[13] Muth J.T., Vogt D.M., Truby R.L., Menguc Y., Kolesky D.B., Wood R.J., Lewis J.A. (2014). Embedded 3D Printing of Strain Sensors within Highly Stretchable Elastomers. Adv. Mater..

[14] Al-Rubaiai M., Tsuruta R., Gandhi U., Wang C., Tan X.B. (2019). A 3D-Printed Stretchable Strain Sensor for Wind Sensing. Smart Mater. Struct..

[15] Kisic M., Blaz N., Zivanov L., Damnjanovic M. (2020). Elastomer Based Force Sensor Fabricated by 3D Additive Manufacturing. AIP Adv..

[16] Kisic M., Blaz N., Zivanov L., Damnjanovic M. Capacitive Force Sensor Fabricated in Additive Technology. Proceedings of the 42nd International Spring Seminar on Electronics Technology (ISSE).

[17] Adamski K., Kawa B., Walczak R. (2018). Inkjet 3D Printed Micropot with Integrated Cantilever-Like Force Sensor for Growing Plant Biological Potential Measurement. Proceedings.

[18] Delamare J., Sanders R., Krijnen G. 3D Printed Biomimetic Whisker-based Sensor with Co-Planar Capacitive Sensing. Proceedings of the 15th IEEE Sensors Conference.

[19] Qu J.T., Wu Q.Y., Clancy T., Liu X.Y. Design and Calibration of 3D-Printed Micro Force Sensors. Proceedings of the 1st International Conference on Manipulation, Automation and Robotics at Small Scales.

[20] Zhao Z., Liu H.G., Xiong K. (2017). Research on Preparation and Performance of a Waist-Shaped Micro Pressure Sensor based on 3D printing Technology. J. Changzhou Univ..

[21] Lucklum F., Dumstorff G. 3D Printed Pressure Sensor with Screen-Printed Resistive Read-Out. Proceedings of the 15th IEEE Sensors Conference.

[22] Joo H., Cho S. (2020). Comparative Studies on Polyurethane Composites Filled with Polyaniline and Graphene for DLP-Type 3D Printing. Polymers.

[23] Han H., Cho S. (2018). Fabrication of Conducting Polyacrylate Resin Solution with Polyaniline Nanofiber and Graphene for Conductive 3D Printing Application. Polymers.

[24] BMF nanoArch M160. http://www.bmftec.cn/zh/print/details/5.

[25] Faller L.M., Granig W., Krivec M., Abram A., Zangl H. (2018). Rapid Prototyping of Force/Pressure Sensors Using 3D- and Inkjet-Printing. J. Micromech. Microeng..

[26] Liu M., Zhang Q., Shao Y., Liu C., Zhao Y. (2019). Research of a Novel 3D Printed Strain Gauge Type Force Sensor. Micromachines.

[27] Al-Chami H. (2010). Inkjet Printing of Transducers. Master of Applied Science Thesis.

[28] Li K.J. (2002). New Sourcebook of Sensor Technology.

[29] Dawoud M., Taha I., Ebeid S.J. (2018). Strain Sensing Behaviour of 3D Printed Carbon Black Filled ABS. J. Manuf. Process..

[30] Zhang B. (2017). Research on Piezoresistive Performance of Nanocarbons/Silicon Rubber Composites based on Conductive Structure Construction. Ph.D. Thesis.

[31] High Temp Resin for Heat Resistance, Material Data Sheet. https://formlabs-media.formlabs.com/datasheets/High_Temp_Technical.pdf.

[32] Wang Y., Liu Z., Yi J., Xue Z. (2011). Study on the Piezoresistive Effect of the Multiwalled Carbon Nanotube Films. Acta Phys. Sin..

[33] He Y., Wu X., Lin H., Yu M. (1996). The Structure and Piezo-Resistance Effect of Hydrogenated Nano-Si Films. Chinses J. Mater. Res..

[34] Ma Q., Xu H., Lei C., Zhang Z., Zhang P., Wu S., Li H. (2018). Effect of Curing Conditions on Curing Rate and Mechanical Strength of Unsaturated Polyester Resin. Guangzhou Archit..

[35] Grimaldi C., Ryser P., Strassler S. (2001). Gauge Factor Enhancement Driven by Heterogeneity in Thick-Film Resistors. J. Appl. Phys..

[36] Li B., Zhao Y., Li C., Cheng R., Sun D., Wang S. (2017). A Differential Resonant Accelerometer with Low Cross-Interference and Temperature Drift. Sensors.

